# Greater and Clinically Meaningful Improvement in Health-Related Quality of Life After Device-Aided Therapy in Younger Patients Earlier Within Advanced Parkinson’s Disease: Real-World Evidence from the DATs-PD GETM Spanish Registry

**DOI:** 10.3390/medsci14060602

**Published:** 2026-09-24

**Authors:** Diego Santos García, Ángela Solleiro Vidal, Lydia López Manzanares, Silvia Martí Martínez, Jéssica González Ardura, Juan Carlos Gómez Esteban, Tania Delgado Ballestero, Guillermo González Ortega, Esther Cubo, Berta Solano, Ana Belén Perona-Moratalla, Sonia Escalante, Ángela Monterde Ortega, Maria Pilar Gil Martín, Roberto López Blanco, Carmen Borrué Fernández, Irene Martínez Torres, Pedro García Ruíz, Isabel Pareés Moreno, Celia Delgado Suárez, DATs-PD GETM Spanish Registry Group

**Affiliations:** 1 Complejo Hospitalario Universitario de A Coruña (CHUAC), 15006 A Coruña, Spain; 2 Grupo de Investigación en Enfermedad de Parkinson y otros Trastornos del Movimiento, INIBIC (Instituto de Investigación Biomédica de A Coruña), 15006 A Coruña, Spain; 3 Hospital San Rafael, 15006 A Coruña, Spain; 4 Fundación Degen, 15006 A Coruña, Spain; 5 Networking Research Center on Neurodegenerative Diseases (CIBERNED), Instituto de Salud Carlos III, 28040 Madrid, Spain; 6 Hospital Universitario La Princesa, 28040 Madrid, Spain; 7 Hospital General Universitario Alicante, 03203 Alicante, Spain; 8 Hospital de Cabueñes, 33394 Gijón, Spain; 9 Hospital Universitario de Cruces, 48903 Bilbao, Spain; 10 Parc Taulí de Sabadell, 08208 Barcelona, Spain; 11 Hospital Universitario de Móstoles, 28040 Madrid, Spain; 12 Hospital Universitario de Burgos, 09006 Burgos, Spain; 13 Department of Neurology, Hospital Josep Trueta, 17007 Girona, Spain; 14 Department of Neurology, Hospital Santa Caterina, 17004 Girona, Spain; 15 Complejo Hospitalario Universitario de Albacete, 03203 Albacete, Spain; 16 Hospital Virgen de la Cinta, 43500 Tortosa, Spain; 17 Hospital Universitario Joan XXIII, 43005 Tarragona, Spain; 18 Hospital Universitari Arnau de Vilanova, 25198 Lleida, Spain; 19 Hospital Universitario Severo Ochoa, 28040 Madrid, Spain; 20 Hospital Universitario Infanta Sofía, 28040 Madrid, Spain; 21 Hospital Universitario La Fe, 46026 Valencia, Spain; 22 Hospital Fundación Jiménez Díaz, 28040 Madrid, Spain; 23 Hospital Universitario Ramón y Cajal, 28040 Madrid, Spain; 24 Servicio de Neurología, Hospital Universitario Son Llàtzer, 07198 Palma de Mallorca, Spain

**Keywords:** advanced Parkinson’s disease, device-aided therapy, health-related quality of life, real-world evidence, treatment timing, younger patients

## Abstract

**Background and objective:** Device-aided therapies (DATs) are established treatments for Parkinson’s disease (PD) with motor fluctuations insufficiently controlled by conventional therapy, but their optimal timing remains uncertain. We investigated whether younger patients earlier within advanced PD (yeaPD) experience greater health-related quality-of-life (HRQoL) improvement after DAT initiation. **Methods:** Data were obtained from the prospective, multicenter DATs-PD GETM Spanish Registry. yeaPD was defined as age ≤65 years, Hoehn and Yahr stage ≤2 in the ON state, and ≤5 years since motor fluctuation onset. HRQoL was assessed using the Parkinson’s Disease Questionnaire Summary Index (PDQ-39SI) at baseline and 6 ± 3 months. **Results:** Of 510 patients, 133 (26.1%) fulfilled yeaPD criteria. Paired data were available for 148 patients (36 yeaPD; 112 non-yeaPD). PDQ-39SI improved from 31.7 ± 13.8 to 21.9 ± 16.0 in yeaPD and from 38.5 ± 15.6 to 34.8 ± 17.1 in non-yeaPD (relative improvements: 30.9% vs. 9.6%; *p* = 0.032). The group-by-time interaction remained significant after adjustment for sex, principal caregiver, DAT type, time since PD diagnosis, and baseline levodopa equivalent daily dose (F = 4.565, *p* = 0.035), and after additionally adjusting for changes in motor and non-motor symptom burden (F = 4.460, *p* = 0.037). Clinically relevant HRQoL improvement occurred in 75.0% versus 53.6% (*p* = 0.023; OR 2.60, 95% CI 1.12–6.03). Mobility and stigmatization remained significant after multiplicity correction. **Conclusions:** DAT initiation while patients remain younger and functionally less advanced was associated with greater, clinically meaningful HRQoL improvement.

## 1. Introduction

Parkinson’s disease (PD) is a progressive neurodegenerative disorder characterized by a combination of motor and non-motor manifestations that increasingly interfere with autonomy and health-related quality of life (HRQoL) [1,2,3]. HRQoL in PD is influenced not only by motor disability but also by non-motor symptoms, fluctuations, mood, cognition, social participation, and loss of independence [4,5,6]. Parkinson’s disease is also an increasingly important public-health challenge, with prevalence rising as populations age and with epidemiological patterns potentially influenced by demographic, environmental, and lifestyle factors [7]. Pathophysiologically, PD reflects progressive dysfunction across dopaminergic and non-dopaminergic networks, producing marked clinical heterogeneity. Disease evolution is likewise heterogeneous: motor phenotypes such as tremor-dominant and postural-instability/gait-difficulty presentations may follow different trajectories, although phenotype classification can change over time and should not be regarded as a fixed biological subtype [8,9]. Although levodopa remains the most effective symptomatic therapy, motor fluctuations and dyskinesias commonly emerge during long-term treatment and can become increasingly difficult to control with conventional oral or transdermal regimens [10].

The transition to advanced Parkinson’s disease (aPD) is not defined by a single milestone. Expert consensus, observational data, and pragmatic screening criteria have therefore been proposed to identify patients whose symptoms are insufficiently controlled and who should be evaluated for device-aided therapies (DATs) [11,12,13]. Available DATs include deep brain stimulation (DBS), continuous subcutaneous apomorphine infusion (CSAI), continuous subcutaneous foslevodopa/foscarbidopa (fLD/fCD), levodopa–carbidopa intestinal gel (LCIG), and levodopa–entacapone–carbidopa intestinal gel (LECIG) [14,15,16]. Candidate selection for advanced therapies also requires individualized assessment of comorbidities and therapy-specific risks. These considerations vary according to the selected DAT [17]. DATs reduce motor fluctuations and can improve non-motor symptoms, activities of daily living, and HRQoL [18,19,20,21]. However, in routine practice these therapies may still be considered relatively late, after substantial disability has developed. This raises a clinically important question: could patients obtain a larger HRQoL benefit if DATs are introduced while they retain greater functional reserve and before treatment-resistant axial, cognitive, or other non-dopaminergic manifestations become dominant? This hypothesis has recently gained support from a post hoc analysis of the randomized phase 3 fLD/fCD trial. Antonini et al. defined a subgroup of ‘younger patients earlier within advanced Parkinson’s disease’ (yeaPD) as patients aged ≤65 years, with Hoehn and Yahr (H&Y) stage ≤2 in the ON state and ≤5 years since motor fluctuations began [22]. Only 26 participants fulfilled these criteria, but treatment effects with fLD/fCD were numerically larger than in the overall trial population across several outcomes, including an approximately 10-point improvement in PDQ-39. The authors specifically called for larger real-world studies to test this concept [22]. Continuous dopaminergic delivery provides a mechanistic rationale for timely treatment by reducing the pulsatile exposure associated with intermittent oral levodopa and by stabilizing motor response [23]. Nevertheless, the yeaPD concept has not been independently evaluated in a large prospective real-world cohort receiving the range of DATs used in contemporary clinical practice.

The DATs-PD GETM Spanish Registry is an ongoing prospective, multicenter clinical registry including patients treated with DATs under routine clinical practice at more than 40 centers in Spain [24]. The present study aimed to compare longitudinal change in HRQoL after DAT initiation between yeaPD and non-yeaPD patients. We hypothesized that yeaPD status would be associated with a greater improvement in PDQ-39SI and a higher probability of achieving a clinically relevant HRQoL response.

## 2. Materials and Methods

### 2.1. Study Design and Population

This study is an observational analysis of prospectively collected data from the DATs-PD GETM Spanish Registry. The registry design has been published previously [24]. Briefly, it is a descriptive, prospective, multicenter, open clinical registry with progressive inclusion of people with PD treated with a DAT under routine clinical practice conditions in more than 40 Spanish centers. For the present analysis, data available through 30 October 2025 were used.

Patients were evaluated before initiation of the DAT (baseline; V0) and at the first follow-up visit scheduled at 6 ± 3 months (V6M). DATs represented in the registry include DBS, CSAI, fLD/fCD, LCIG, and LECIG. Treatment selection was made by the treating neurologist according to routine clinical practice and individual patient characteristics.

### 2.2. Definition of yeaPD

Patients were classified as yeaPD when all three criteria proposed by Antonini et al. [22] were fulfilled: (1) age ≤65 years at DAT initiation; (2) H&Y stage ≤2 during the ON state; and (3) ≤5 years since onset of motor fluctuations. Patients who did not meet all three criteria were classified as non-yeaPD.

### 2.3. Clinical Assessments

Baseline variables included age at DAT initiation, age at PD diagnosis, disease duration, sex, motor phenotype, presence of a principal caregiver, previous and current PD treatments, previous on-demand or device-aided therapies, levodopa equivalent daily dose (LEDD) [25], dopamine agonist equivalent daily dose, body mass index, previous psychosis, cognitive impairment, duration of motor fluctuations, H&Y stage, UPDRS Part III scores, daily OFF time, motor symptom (MS) burden (MSs [Motor Symptoms score]), dyskinesia burden (dyskinesia score), non-motor symptom (NMS) burden (Non-Motor Symptoms score [NMSs]), PDQ-39SI, EUROHIS-QOL8, Schwab and England Activities of Daily Living Scale (ADLS), and level of independence. MS, NMS, and dyskinesia burden scores were calculated according to the registry methodology and previous publications cited in the source dataset [24,26]. A standardized, comprehensive comorbidity index was not available for all participants in the analytic dataset; therefore, individual comorbidities could not be compared reliably between yeaPD and non-yeaPD groups and are acknowledged as a limitation.

### 2.4. Health-Related Quality of Life

HRQoL was assessed using the 39-item Parkinson’s Disease Questionnaire (PDQ-39), a validated PD-specific patient-reported outcome measure [27,28]. The PDQ-39 Summary Index (PDQ-39SI) ranges from 0 to 100, with higher scores representing worse HRQoL. The instrument contains eight domains: mobility, activities of daily living (ADL), emotional well-being, stigmatization, social support, cognition, communication, and bodily discomfort.

The main outcome was longitudinal change in PDQ-39SI from V0 to V6M according to yeaPD status. Domain-specific relative changes were considered exploratory secondary outcomes. A responder analysis evaluated the proportion of patients achieving a clinically relevant improvement in PDQ-39SI. Based on the published PDQ-39SI minimal clinically important difference (MCID), a decrease of 4.72 points has been proposed as the threshold for clinically important improvement [29].

### 2.5. Statistical Analysis

Continuous variables were summarized as mean ± standard deviation or median [25th–75th percentile], as appropriate, and categorical variables as percentages. Baseline comparisons between yeaPD and non-yeaPD were performed using Student’s *t*-test, Mann–Whitney–Wilcoxon tests, and chi-square tests according to variable type and distribution. Within-group PDQ-39SI changes were assessed using paired non-parametric testing. Relative changes in PDQ-39 domains were compared using Mann–Whitney–Wilcoxon tests. To evaluate potential attrition bias, baseline characteristics were compared between participants with complete paired V0–V6M PDQ-39SI data and those without complete paired data.

To test whether longitudinal PDQ-39SI trajectories differed between groups after adjustment, repeated-measures analysis of variance (RM-ANOVA) models were fitted with time as the within-subject factor and yeaPD status as the grouping factor. Model 1 included sex, presence of a principal caregiver, DAT type, time since PD diagnosis, baseline LEDD, and yeaPD status. The key effect of interest was the Time × group interaction. Model 2 additionally included change from baseline to follow-up in MS burden and NMS burden. Because changes in MS and NMS burden occur after DAT initiation and may lie on the pathway linking treatment to HRQoL, Model 1 was considered the principal adjusted model for interpretation, whereas Model 2 was treated as a sensitivity analysis. In response to the multicenter nature of the registry, an additional sensitivity analysis modeled absolute PDQ-39SI change (V6M minus V0) with the same baseline covariates as Model 1 and cluster-robust standard errors at the recruiting-center level; recruiting center was derived from the center prefix of the study identifier. A further sensitivity model additionally included baseline cognitive status (no impairment, mild cognitive impairment, or dementia).

For the responder analysis, proportions were compared using Pearson’s chi-square test. From the reported counts, the unadjusted odds ratio (OR), risk ratio (RR), and absolute risk difference were calculated with 95% confidence intervals. All tests were two-sided. A *p* value < 0.05 was considered nominally significant. For the eight exploratory PDQ-39 domain comparisons, a Bonferroni-adjusted threshold of *p* < 0.00625 was additionally applied.

### 2.6. Ethics

The DATs-PD GETM Spanish Registry is conducted in accordance with the Declaration of Helsinki and applicable Spanish legislation. Written informed consent was obtained from all participants. The study protocol was approved on 2 April 2024, by the Galicia Clinical Research Ethics Committee (protocol code: 2024/109).

## 3. Results

### 3.1. Study Population and Baseline Characteristics

A total of 510 patients treated with a DAT were included in the baseline analysis. Mean age at DAT initiation was 66.5 ± 9.7 years, mean age at PD diagnosis was 56.2 ± 10.6 years, mean disease duration was 10.4 ± 6.5 years, and 59.2% were male.

Of the 510 patients, 214 (42.0%) were ≤65 years old, 402 (78.8%) had H&Y-ON ≤2, and 352 (69.0%) had experienced motor fluctuations for ≤5 years. A total of 133 patients (26.1%) fulfilled all three yeaPD criteria; 377 (73.9%) were classified as non-yeaPD.

By definition and clinical profile, yeaPD patients were substantially younger and had shorter disease and fluctuation duration. They also showed a lower MS and NMS burden, greater functional independence, and better baseline HRQoL. Among participants with a baseline PDQ-39SI available for the baseline cross-sectional analysis, PDQ-39SI was 32.9 ± 14.9 in yeaPD and 39.3 ± 15.8 in non-yeaPD (*p* = 0.001). These values refer to the broader baseline-available sample and therefore differ slightly from the baseline values reported below for the 148-patient paired longitudinal subset. Detailed baseline characteristics are shown in Table 1.

### 3.2. Longitudinal Change in PDQ-39SI

Participant flow is summarized in Figure 1. Of the 510 DAT-treated participants included in the baseline cohort, PDQ-39SI was available at baseline in 339 and at V6M in 170. Complete paired V0 and V6M PDQ-39SI assessments were available for 148 patients (36 yeaPD and 112 non-yeaPD). Thus, 191 of the 339 participants with a baseline PDQ-39SI did not contribute complete paired data, while 22 participants with a V6M PDQ-39SI lacked an available baseline PDQ-39SI. Mean follow-up occurred 188.6 ± 72.9 days after baseline. In non-yeaPD patients, PDQ-39SI decreased from 38.5 ± 15.6 at baseline to 34.8 ± 17.1 at V6M (within-group *p* = 0.003), corresponding to a relative improvement of 9.6%. In yeaPD patients, PDQ-39SI decreased from 31.7 ± 13.8 to 21.9 ± 16.0 (within-group *p* = 0.001), corresponding to a relative improvement of 30.9%. The relative improvement was significantly greater in yeaPD than in non-yeaPD (*p* = 0.032; Figure 2A). In absolute terms, the mean decrease was approximately 9.8 PDQ-39SI points in yeaPD compared with 3.7 points in non-yeaPD.

An attrition analysis compared the 148 participants with complete paired PDQ-39SI data with the 362 participants without complete paired data. The groups were similar in age at DAT initiation (67.1 ± 9.2 vs. 66.3 ± 10.0 years; *p* = 0.387), sex (64.2% vs. 57.2% male; *p* = 0.144), yeaPD prevalence (24.3% vs. 26.8%; *p* = 0.564), time since PD diagnosis (11.1 ± 6.2 vs. 10.1 ± 6.7 years; *p* = 0.097), duration of motor fluctuations (4.7 ± 2.4 vs. 4.4 ± 2.5 years; *p* = 0.167), H&Y-ON stage (2.14 ± 0.58 vs. 2.19 ± 0.65; *p* = 0.344), baseline NMS burden (12.5 ± 6.7 vs. 12.1 ± 7.3; *p* = 0.566), baseline PDQ-39SI among those with an available baseline assessment (36.9 ± 15.4 vs. 38.4 ± 16.1; *p* = 0.386), cognitive impairment (23.1% vs. 25.3%; *p* = 0.611), and DAT distribution (*p* = 0.408). Baseline MS burden was modestly higher in participants with complete paired data (16.3 ± 5.9 vs. 14.8 ± 6.0; *p* = 0.010). These findings reduce, but do not eliminate, concern regarding selection bias from incomplete longitudinal data.

**Figure 2:**
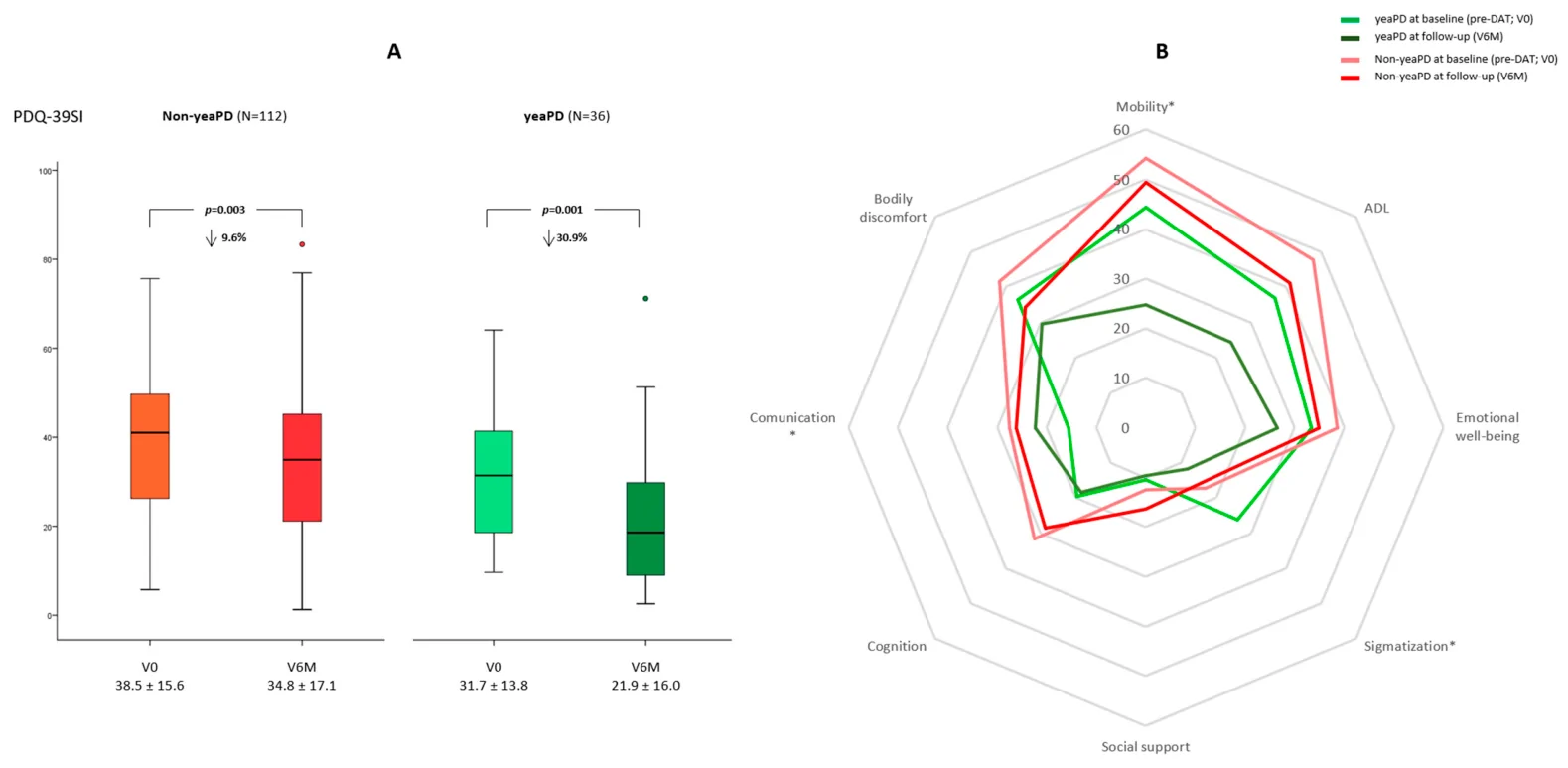
The change in HRQoL after DAT initiation according to yeaPD status. (**A**) PDQ-39SI at baseline (pre-DAT; V0) and follow-up at 6 ± 3 months (V6M) in yeaPD and non-yeaPD patients. The data are shown as box plots. (**B**) Mean values of the eight PDQ-39 domains at V0 and V6M in yeaPD and non-yeaPD patients. The asterisks denote domain-level differences highlighted in the source figure. Domain-level inference is detailed in Table 2, including multiplicity-adjusted interpretation. HRQoL, health-related quality of life; DAT, device-aided therapy; PDQ-39SI, 39-item Parkinson’s Disease Questionnaire Summary Index; yeaPD, younger patients earlier within advanced Parkinson’s disease.

### 3.3. Adjusted Group-by-Time Interaction

The RM-ANOVA confirmed a significant differential longitudinal trajectory of PDQ-39SI according to yeaPD status. In Model 1, adjusted for sex, presence of a principal caregiver, DAT type, time since PD diagnosis, and baseline LEDD, the Time × group interaction was significant (F(1,108) = 4.565, *p* = 0.035, partial η^2^ = 0.041).

The interaction remained significant in Model 2 after additional adjustment for change in MS and NMS burden (F(1,98) = 4.460, *p* = 0.037, partial η^2^ = 0.044). Change in MS burden was itself associated with PDQ-39SI change in Model 2 (Time × change in MS burden: F = 6.827, *p* = 0.010, partial η^2^ = 0.065), whereas change in NMS burden was not (*p* = 0.673). The persistence of the group-by-time interaction in Model 2 was considered a sensitivity finding because symptom changes may partially mediate the HRQoL response.

The center-clustered sensitivity analysis included 115 participants with complete covariate data from 22 recruiting centers. After adjustment for sex, principal caregiver, DAT type, time since PD diagnosis, and baseline LEDD, yeaPD status remained associated with a greater reduction in PDQ-39SI (adjusted between-group difference in change −7.62 points, cluster-robust SE 2.29, 95% CI −12.39 to −2.86; *p* = 0.003). When baseline cognitive status was added, the association remained significant (adjusted difference −6.48 points, cluster-robust SE 2.68, 95% CI −12.06 to −0.90; *p* = 0.025). Baseline cognitive status itself was not significantly associated with PDQ-39SI change in this sensitivity model (*p* = 0.259).

### 3.4. Clinically Relevant Improvement in HRQoL

A clinically relevant improvement in PDQ-39SI was observed in 27 of 36 yeaPD patients (75.0%) compared with 60 of 112 non-yeaPD patients (53.6%; Pearson chi-square *p* = 0.023) (Figure 3). Overall, 87 of 148 patients (58.8%) achieved a clinically relevant improvement.

The absolute difference in responder rate was 21.4 percentage points (95% CI 4.5–38.3). yeaPD patients had a 40% higher probability of response than non-yeaPD patients (RR 1.40, 95% CI 1.08–1.81) and 2.60-fold higher odds of response (OR 2.60, 95% CI 1.12–6.03).

### 3.5. PDQ-39 Domains

The largest relative improvements among yeaPD patients were observed in stigmatization (−54.9%), mobility (−44.2%), and ADL (−33.9%) (Table 3). Between-group differences were nominally significant for mobility (−44.2% vs. −9.1%; *p* = 0.001), stigmatization (−54.9% vs. −9.0%; *p* < 0.0001), and communication (+43.2% vs. −4.8%; *p* = 0.030). After Bonferroni correction for eight domain comparisons (α = 0.00625), only mobility and stigmatization remained statistically significant; the communication finding should therefore be interpreted as nominal and exploratory.

## 4. Discussion

The principal finding of this prospective multicenter real-world study is that patients classified as younger and earlier within aPD experienced a substantially greater improvement in HRQoL after DAT initiation than other DAT-treated patients. At approximately 6 months, PDQ-39SI improved by 30.9% in yeaPD versus 9.6% in non-yeaPD. Importantly, the difference was not limited to an unadjusted comparison: the group-by-time interaction remained significant in the principal adjusted RM-ANOVA model and in a sensitivity model that additionally accounted for changes in motor and non-motor symptom burden.

The finding was also clinically interpretable at the individual patient level. Three of four yeaPD patients achieved a clinically relevant improvement in PDQ-39SI compared with slightly more than one of two non-yeaPD patients. The absolute difference in responder rate was 21.4 percentage points, with an unadjusted OR of 2.60. Thus, the observed association is supported by complementary continuous, longitudinal, and responder analyses rather than by a single statistical comparison. The magnitude of the mean PDQ-39SI improvement in yeaPD patients was approximately 9.8 points. Horváth et al. estimated an MCID of −4.72 points for PDQ-39SI improvement [29], suggesting that the mean change in yeaPD was about twice this threshold, whereas the mean change in non-yeaPD (approximately −3.7 points) did not reach it. This comparison should be interpreted at the group level, but it reinforces the clinical relevance of the responder analysis.

Our results provide independent real-world support for the hypothesis generated by Antonini et al. [22]. In their post hoc analysis, only 26 patients met the yeaPD criteria, but fLD/fCD produced an approximately 9.9-point PDQ-39 improvement versus 1.9 points with oral levodopa/carbidopa. The near-identical magnitude of PDQ-39 improvement in the present yeaPD cohort is notable because our population was treated under routine practice conditions and included different DATs rather than a single infusion therapy. The novelty of the present study lies in evaluating the yeaPD construct outside the original post hoc trial setting, in a prospective multicenter real-world registry that includes the spectrum of DAT modalities used in contemporary practice. In addition, the analysis combines continuous PDQ-39SI change, adjusted longitudinal modeling, a clinically meaningful responder analysis, and domain-level outcomes. These features extend the original hypothesis while remaining observational and non-causal.

Evidence from other DATs is broadly compatible with the hypothesis that treatment timing may be associated with outcome. LCIG has demonstrated sustained improvements in motor fluctuations, non-motor symptoms, and quality of life in randomized and real-world studies [30,31,32]. Post hoc analysis of GLORIA suggested that activities of daily living may improve particularly in younger patients and those treated after shorter disease duration [33], while earlier multicenter work showed clinically relevant improvement in non-motor symptoms and quality of life with continuous intrajejunal levodopa [34]. Similarly, the EuroInf study and TOLEDO trial support clinically meaningful benefits of continuous apomorphine infusion in advanced PD [35,36], and a prospective study specifically examining CSAI in an early phase of aPD reported marked HRQoL improvement over 6 months [37]. The same general principle is supported by the DBS literature. The EARLYSTIM trial demonstrated that subthalamic neurostimulation improved quality of life compared with medical therapy in patients with relatively early motor complications [38]. Earlier randomized trials established the efficacy of DBS in more advanced populations [39,40]. Although surgical and infusion therapies have different mechanisms and selection criteria, these data provide context for investigating whether clinical stage at DAT initiation is associated with subsequent outcomes, without establishing a causal effect of earlier treatment.

The expanding DAT landscape further increases the importance of timely identification. Continuous subcutaneous fLD/fCD has demonstrated efficacy for reducing OFF time and increasing ON time without troublesome dyskinesia in randomized and long-term phase 3 studies [41,42], with practical guidance now available for outpatient initiation and maintenance [43]. LECIG offers another continuous levodopa-based option with clinical and pharmacokinetic evidence supporting its use [44,45]. Contemporary reviews emphasize that continuous levodopa therapies can improve fluctuations and, in appropriately selected patients, quality of life [46].

Several hypotheses could potentially explain the larger HRQoL gains observed in yeaPD patients, but none of these mechanisms was directly measured in the present study and they should therefore be regarded as exploratory interpretations. First, younger patients with shorter fluctuation duration may have a larger proportion of disability driven by levodopa-responsive fluctuations rather than treatment-resistant axial, cognitive, or other non-dopaminergic manifestations. Second, greater baseline independence may provide more opportunity to translate better motor control into participation in work, family, social, and daily activities. Third, more stable dopaminergic stimulation could theoretically be more beneficial while the therapeutic window remains relatively preserved [23]. These explanations require prospective mechanistic evaluation. The domain results are compatible with, but do not prove, these hypotheses. Mobility improved by 44.2% in yeaPD compared with 9.1% in non-yeaPD, while stigmatization improved by 54.9% versus 9.0%; both survived Bonferroni correction. Mobility is a direct and biologically plausible target of DATs. The pronounced improvement in stigmatization is particularly interesting because stigma reflects the social visibility and consequences of PD, including fluctuations, dyskinesia, gait impairment, loss of autonomy, and reduced participation. Younger and more independent patients may experience greater psychosocial improvement when motor function becomes more predictable; this remains a hypothesis because the relevant mediators were not directly measured. The communication signal was only nominally significant and should not be interpreted as confirmatory.

An important methodological strength is that the Time × group interaction remained significant after adjustment for several relevant baseline characteristics, including DAT type, disease duration, and baseline LEDD. Model 1 is the most informative adjusted analysis because it focuses on baseline confounding. Model 2 additionally adjusted for changes in motor and non-motor symptom burden; because these changes occur after DAT initiation and could mediate improvement in HRQoL, this model may partly overadjust the association. Nevertheless, persistence of the group-by-time interaction in Model 2 suggests that the differential HRQoL trajectory was not completely explained by measured motor or non-motor symptom changes.

On another note, cognitive status deserves particular consideration because baseline cognitive impairment was more frequent in non-yeaPD than yeaPD patients. Cognition can independently influence HRQoL, functional independence, treatment selection, and completion of patient-reported outcomes. Importantly, in the additional center-clustered sensitivity model that incorporated baseline cognitive status, yeaPD status remained associated with greater PDQ-39SI improvement (adjusted difference −6.48 points; 95% CI −12.06 to −0.90; *p* = 0.025), whereas baseline cognitive status was not independently associated with change (*p* = 0.259). This sensitivity analysis reduces, although it does not eliminate, concern that the observed group difference was driven by baseline cognitive imbalance.

These findings have practical implications. They should not be interpreted as evidence that every younger patient with motor fluctuations should receive a DAT immediately. DAT selection remains individualized and must consider the predominant symptoms, cognition, psychiatric status, comorbidity, caregiver support, patient preferences, and the benefit–risk profile of each therapy [14,15,16,17,18,19,20]. Rather, the results support timely referral and discussion of DAT options once conventional therapy no longer provides adequate control, rather than systematically postponing evaluation until severe disability has developed.

### Strengths and Limitations

The study has several strengths: prospective data collection within a national multicenter registry; inclusion of more than 40 centers; use of a yeaPD definition established independently in a previous clinical-trial analysis; focus on a patient-reported outcome; inclusion of different DATs; and confirmation of the unadjusted finding using adjusted longitudinal and responder analyses.

Several limitations must be emphasized. First, the study is observational, non-randomized, and lacks a non-DAT comparator; therefore, the observed association between yeaPD status and HRQoL change cannot establish that earlier DAT initiation caused greater benefit. yeaPD and non-yeaPD patients differ substantially by definition and clinical phenotype, and residual confounding remains possible despite adjustment. Second, complete paired PDQ-39SI data were available for only 148 of 510 baseline participants. This substantial attrition limits generalizability. The formal attrition analysis showed broadly similar baseline characteristics between participants with and without complete paired data, although MS burden was modestly higher in completers; selection bias therefore cannot be excluded. Third, the multicenter design may introduce within-center correlation. A center-clustered sensitivity analysis across 22 centers yielded a consistent association, but the number of participants within some centers was limited and center-level effects cannot be fully excluded. Fourth, some baseline variables had missing data, as expected in a real-world registry. Fifth, different DATs were pooled, introducing treatment heterogeneity; DAT type was adjusted for, but treatment-specific analyses were underpowered. Sixth, the yeaPD construct combines age, disease stage, and duration of fluctuations, so the present analysis cannot determine which component is most predictive of response. Seventh, baseline cognitive impairment differed between groups; the association persisted after adjustment for cognitive status, but residual cognitive confounding remains possible. Eighth, a standardized comprehensive comorbidity index was not available for all participants. Ninth, the proposed biological and functional explanations were not directly measured and remain hypotheses. Tenth, domain analyses were exploratory and required multiplicity correction. Finally, follow-up was limited to approximately 6 months; longer-term data are required to determine whether the differential HRQoL association is sustained.

## 5. Conclusions

In this prospective multicenter real-world analysis, approximately one quarter of patients initiating a DAT fulfilled criteria for being younger and earlier within advanced PD. yeaPD patients showed a substantially greater improvement in PDQ-39SI than non-yeaPD patients, and the differential longitudinal trajectory remained significant after adjustment for relevant clinical variables. In addition, 75% of yeaPD patients achieved a clinically relevant HRQoL improvement compared with 53.6% of non-yeaPD patients.

These findings provide real-world external support for the yeaPD hypothesis and show that yeaPD status was associated with a larger and more clinically meaningful HRQoL improvement after DAT initiation. The association remained evident in sensitivity analyses addressing center clustering and baseline cognitive status, while the attrition analysis showed generally comparable baseline profiles between participants with and without complete paired data. Nevertheless, the results do not establish a causal benefit of earlier DAT initiation. Future work should include longer follow-up, independent replication, treatment-specific analyses, more comprehensive adjustment for comorbidity and center effects, prospective validation of the individual components of the yeaPD construct, and studies including an appropriate comparator group.

## Figures and Tables

**Figure 1:**
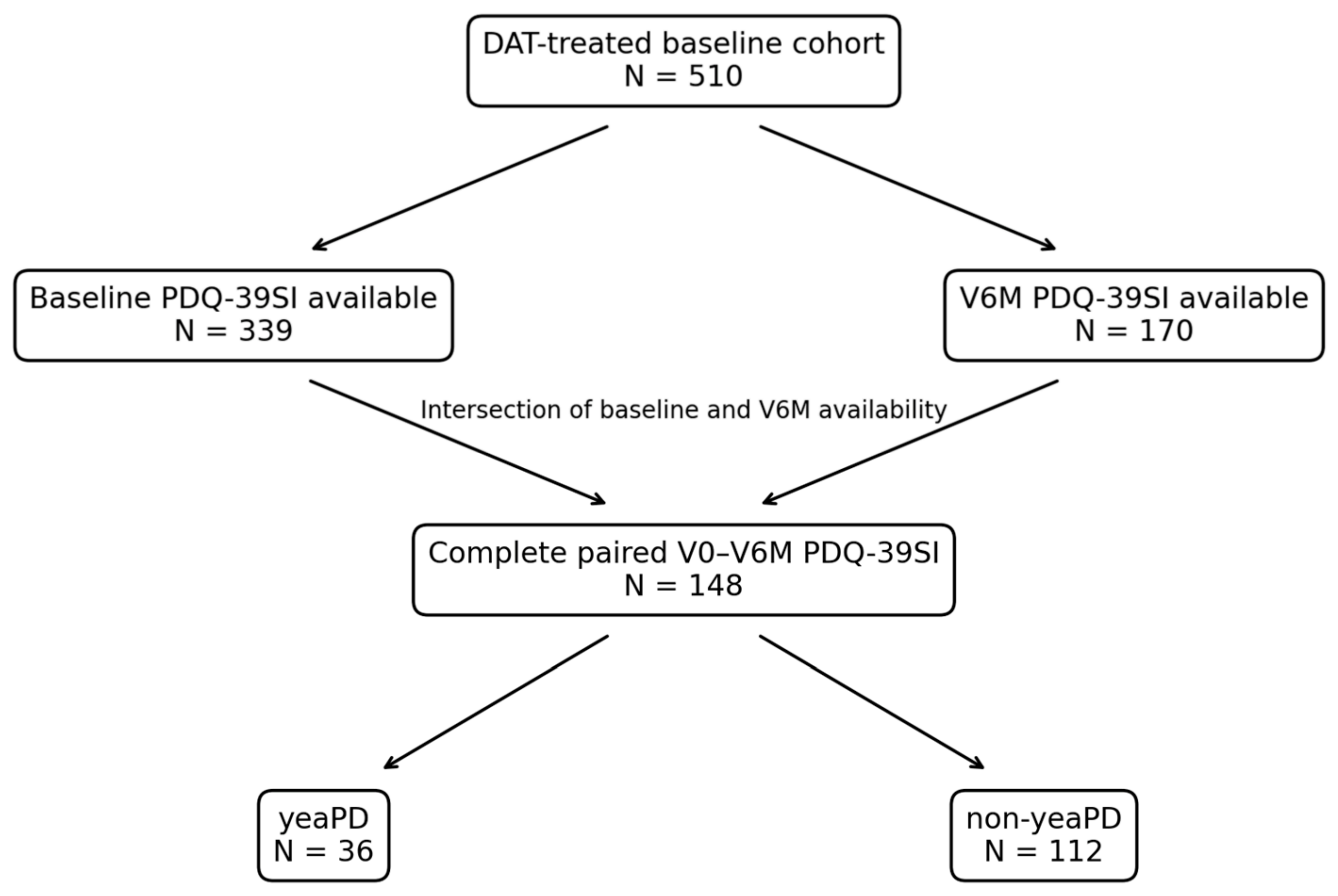
STROBE-style participant flow for the PDQ-39SI analyses. Of 510 DAT-treated participants, baseline PDQ-39SI was available in 339 and V6M PDQ-39SI in 170; 148 had complete paired V0–V6M data and constituted the longitudinal cohort (36 yeaPD and 112 non-yeaPD). PDQ-39SI, 39-item Parkinson’s Disease Questionnaire Summary Index; V0, baseline; V6M, 6 ± 3-month follow-up.

**Figure 3:**
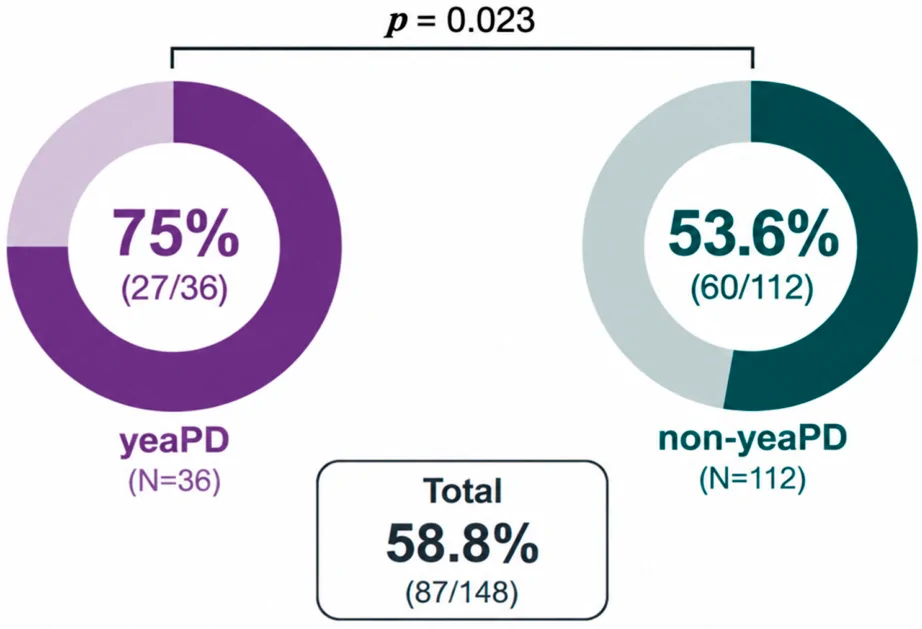
Clinically relevant improvement in HRQoL according to yeaPD status. The figure shows the proportion of patients achieving a clinically relevant improvement in PDQ-39SI from baseline to V6M in yeaPD and non-yeaPD groups. The comparison shown uses Pearson’s chi-square test (*p* = 0.023). HRQoL, health-related quality of life; PDQ-39SI, 39-item Parkinson’s Disease Questionnaire Summary Index; yeaPD, younger patients earlier within advanced Parkinson’s disease.

**Table 1:** PD-related variables at baseline (before initiation of a DAT) in yeaPD vs. non-yeaPD patients from the DATs-PD GETM Spanish Registry.

Variable	All Cohort N = 510	yeaPD N = 133	Non-yeaPD N = 377	*p*
Age at initiation of DAT	66.5 ± 9.7	56.7 ± 6.3	69.9 ± 8.3	**<0.0001**
Age at PD diagnosis	56.2 ± 10.6	49.5 ± 6.4	58.5 ± 10.8	**<0.0001**
Years since PD diagnosis	10.4 ± 6.5	7.4 ± 3.5	11.5 ± 7	**<0.0001**
Males (%)	59.2	71.4	54.9	**0.001**
Motor phenotype (%)				0.390
Tremor dominant	33	36.9	31.8	
Indeterminate	33	34.3	32.6	
PIGD	34	28.8	35.6	
Principal caregiver (%)	52.8	28.8	61.2	**<0.0001**
Treatment for PD (%)				
MAO-B inhibitor	66.7	72.3	64.8	0.071
COMT inhibitor	51.7	49.2	52.6	0.290
Dopamine agonist	54.4	57.7	53.3	0.221
Amantadine	26.3	32.1	24.3	0.055
Previous ODT	46.3	48.5	45.6	0.322
Previous DAT	20.6	14.6	22.7	**0.031**
LEDD (mg)	1230.3 ± 558.2	1126.3 ± 519.8	1265.5 ± 567.2	**0.037**
DAEDD (mg)	106.8 ± 125.9	109.2 ± 126.5	106.0 ± 125.9	0.806
BMI	26.2 ± 3.8	25.1 ± 2.1	26.6 ± 4.2	0.311
Previous psychosis (%)	11.2	7.6	12.5	0.085
Cognitive impairment (%)				**<0.0001**
MCI (%)	22.4	10.1	26.8	
Dementia (%)	2.2	0	3.0	
Time with fluctuations (y)	4.5 ± 2.4	3.1 ± 1.3	4.9 ± 2.6	**<0.0001**
H&Y—OFF	3 [2, 4]	3 [2, 3]	3 [2, 4]	**<0.0001**
H&Y—ON	2 [2, 2]	2 [2, 2]	2 [2, 3]	**<0.0001**
UPDRS—III—OFF	38.0 ± 12.5	34.9 ± 11.3	39.2 ± 12.7	**0.002**
UPDRS—III—ON	20.2 ± 10.9	15.6 ± 8.8	21.9 ± 11.2	**<0.0001**
Daily OFF time (h)	5.3 ± 2.9	5.1 ± 2.8	5.4 ± 2.9	0.235
MS burden	15.3 ± 6.0	13.3 ± 5.5	16.0 ± 6.0	**<0.0001**
Dyskinesia score	2.7 ± 2.1	2.5 ± 2.0	2.7 ± 2.1	0.262
NMS burden	12.3 ± 7.1	9.4 ± 6.1	13.3 ± 7.2	**<0.0001**
PDQ-39SI	37.7 ± 15.8	32.9 ± 14.9	39.3 ± 15.8	**0.001**
EUROHIS-QOL8	22.4 ± 5.0	22.7 ± 5.3	22.3 ± 4.9	0.574
ADLS—OFF	57.2 ± 24.1	66.1 ± 20.5	54.1 ± 24.6	**<0.0001**
ADLS—ON	81.4 ± 17.7	89.2 ± 12.3	78.6 ± 18.5	**<0.0001**
Daily living activities (%)				**<0.0001**
Independence	62.9	83.1	55.9	
Dependence for IADL	23.4	13.8	26.7	
Dependence for BADL	13.7	3.1	17.4	

**Note.** The results are percentages, mean ± SD, or median [p25, p75]. Chi-square, Student’s *t*-test, and/or Mann–Whitney–Wilcoxon tests were applied according to variable type and distribution. Information was not available for every variable in all patients: the lowest sample sizes were N = 37 for BMI, N = 310 for UPDRS-III-ON, N = 332 for EUROHIS-QOL8, N = 339 for PDQ-39SI, N = 403 for LEDD, N = 408 for UPDRS-III-OFF, N = 425 for ADLS-OFF, and N = 428 for ADLS-ON; sample size was >90% for the remaining variables. Bold *p* values indicate *p* < 0.05. DAT, device-aided therapy; ODT, on-demand therapy; LEDD, levodopa equivalent daily dose; DAEDD, dopamine agonist equivalent daily dose; BMI, body mass index; MCI, mild cognitive impairment; H&Y, Hoehn and Yahr; UPDRS, Unified Parkinson’s Disease Rating Scale; MS, motor symptoms; NMS, non-motor symptoms; PDQ-39SI, 39-item Parkinson’s Disease Questionnaire Summary Index; ADLS, Schwab and England Activities of Daily Living Scale; IADL, instrumental activities of daily living; BADL, basic activities of daily living.

**Table 2:** Repeated-measures ANOVA for change in PDQ-39 Summary Index according to yeaPD status.

Within-Subject Term	Model 1 F	Model 1 *p* Value	Model 1 Partial η^2^	Model 2 F	Model 2 *p* Value	Model 2 Partial η^2^
Time	1.299	0.257	0.012	0.216	0.643	0.002
Time × sex	1.132	0.290	0.010	0.611	0.436	0.006
Time × principal caregiver	0.976	0.325	0.009	0.262	0.610	0.003
Time × DAT type	0.672	0.414	0.006	0.076	0.784	0.001
Time × time since PD diagnosis	0.110	0.740	0.001	0.259	0.612	0.003
Time × baseline LEDD	0.622	0.432	0.006	0.157	0.693	0.002
Time × change in MS burden	—	—	—	6.827	**0.010**	0.065
Time × change in NMS burden	—	—	—	0.179	0.673	0.002
Time × group (yeaPD vs. non-yeaPD)	4.565	**0.035**	0.041	4.460	**0.037**	0.044

**Note.** Dependent variable: PDQ-39SI measured at baseline and V6M. Model 1 included sex, principal caregiver, DAT type, time since PD diagnosis, baseline LEDD, and yeaPD status; error df = 108. Model 2 additionally included change in MS and NMS burden; error df = 98. The Time × group term tests whether longitudinal PDQ-39SI change differs between yeaPD and non-yeaPD. Bold *p* values indicate *p* < 0.05.

**Table 3:** Relative change in PDQ-39SI domains from baseline to V6M in yeaPD vs. non-yeaPD patients.

PDQ-39 Domain	All Cohort N = 148	yeaPD N = 36	Non-yeaPD N = 112	*p*
Mobility	−16.4	−44.2	−9.1	**0.001**
ADL	−18.0	−33.9	−14.1	0.316
Emotional well-being	−11.9	−20.7	−9.5	0.802
Stigmatization	−24.2	−54.9	−9.0	**<0.0001**
Social support	+22.9	−6.6	+30.8	0.289
Cognition	−9.1	−6.1	−9.6	0.838
Communication	+2.5	+43.2	−4.8	**0.030**
Bodily discomfort	−17.9	−19.0	−17.7	0.744

**Note.** Values are relative change (%) from V0 to V6M. Mann–Whitney–Wilcoxon tests compared yeaPD with non-yeaPD. Bold *p* values indicate *p* < 0.05. Communication result (*p* = 0.030) is nominally significant but does not survive multiplicity correction. ADL, activities of daily living.

## Data Availability

The data presented in this study are available on request from the corresponding author, because the data are not publicly available due to privacy or ethical restrictions.

## Abbreviations

ADL, activities of daily living; aPD, advanced Parkinson’s disease; DBS, deep brain stimulation; CSAI, continuous subcutaneous apomorphine infusion; fLD/fCD, subcutaneous foslevodopa/foscarbidopa; H&Y, Hoehn & Yahr; LCIG, levodopa–carbidopa intestinal gel; LECIG, levodopa–entacapone–carbidopa intestinal gel; LEDD, levodopa equivalent daily dose; HRQoL, health-related quality of life; MCID, minimal clinically important difference; MS, motor symptoms; MSs, Motor Symptoms score; NMS, non-motor symptoms; NMSs, Non-Motor Symptoms score; PD, Parkinson’s disease; PDQ-39SI, 39-item Parkinson’s Disease Questionnaire Summary Index; UPDRS, Unified Parkinson’s Disease Rating Scale.

## Appendix A

DATs-PD GETM Spanish Registry STUDY GROUP (only members who participated with available data for this analysis are included here).

Adarmes Gómez AD, Aldaz A, Alonso Modino D, Álvarez Sauco M, Ávila Rivera A, Baviera-Muñoz R, Belmonte S, Blázquez Estrada M, Caballero Sánchez L, Caballol Pons N, Cabo López I, Campins Romeu M, Campo Caballero D, Cantarero Duque S, Cañada Lahoz E, Cardoso Calo N, Carrillo García F, Casas Peña E, Castaño García B, Castellano Guerrero AM, Castrillo Sanz A, Cerdán Santacruz DM, Cores Bartolomé C, Cots Foraster A, Cubo Delgado E, del Álamo Criado C, Delgado Ballestero T, Escalante Arroyo S, Escamilla Sevilla F, Fanjul Arbos S, Feliz Feliz C, Fernández-Pajarín G, Fernández Revuelta A, Fernández Valle T, Freire Álvarez E, Gamo González E, García Cimadevilla S, García Fernández C, García Herruzo A, García Ramos R, García Ruíz Espiga P, Garrote Espina L, Gil Villar MP, Gómez Esteban JC, Gómez López de San Román C, Gómez Mayordomo V, Gómez Rapela C, González MV, González Ardura J, González-Ortega G, González Rojas N, Hernández Javier C, Hernández Vara J, Jesús Maestre S, Legarda Ramírez I, López Ariztegui N, López Blanco R, López Dominguez D, López Manzanares L, López Valdés E, Lorenzo Barreto P, Lorenzo Brito JM, Lozano D, Lozano Veiga S, Macías García D, Mandrá MA, Madrid Navarro CJ, Marcos Conca R, Martí Martínez S, Martín García R, Martínez Castrillo JC, Martínez Miguel C, Martínez-Torres I, Mata Álvarez Santullano M, Mauri Fabrega L, Méndez Guerrero A, Mendoza Rodríguez A, Mir Rivera P, Mondragón Rezola E, Morales Casado MI, Morata-Martínez C, Muñoz Delgado L, Muro I, Navarro Mocholi E, Novo Ponte S, Ojeda Lepe E, Pardina Vilella L, Pareés Moreno I, Paz González JM, Peral Quirós A, Pérez Calvo C, Pérez Rangel D, Perona Moratalla A, Planas-Ballvé A, Prendes Fernández P, Quibus Requena L, Rábano Suárez P, Ramírez J, Rivero de Aguilar Pensado A, Rojas Pérez ME, Ribacoba Díaz C, Romero Fábrega JC, Ruíz López M, Ruíz Martínez J, Samaniego Vinueza LB, San Eufrasio Martínez M, Sánchez Alonso P, Sánchez Ferro A, Sánchez Rodríguez A, Sancho Saldana A, Santos-García D, Sastre Bataller I, Solano Vila B, Solleiro Vidal A, Suárez San Martín E, Tabar Comellas G, Tijero Merino B, Triguero Cueva L, Valero García MF, Vela L, Vinagre Aragón A, Vivas Villacampa L, Vives Pastor B, Yáñez Baña R.

**First Name**	**Last Name**	**Center**
Diego	Santos García	Complejo Hospitalario Universitario de A Coruña
Jose Manuel	Paz González	Complejo Hospitalario Universitario de A Coruña
Carlos	Cores Bartolomé	Complejo Hospitalario Universitario de A Coruña
Ángela	Solleiro Vidal ^2^	Complejo Hospitalario Universitario de A Coruña
Lucia Belen	Samaniego Vinueza	Complejo Hospitalario Universitario de A Coruña
Noelia	Cardoso Calo	Complejo Hospitalario Universitario de A Coruña
María	Álvarez Sauco	Hospital General Universitario de Elche
Eric	Freire Álvarez	Hospital General Universitario de Elche
Juan Carlos	Martínez Castrillo	Hospital Universitario Ramón y Cajal
Isabel	Pareés Moreno	Hospital Universitario Ramón y Cajal
Samira	Fanjul Arbos	Hospital Universitario Ramón y Cajal
Ana Belén	Perona Moratalla	Complejo Hospitalario Universitario de Albacete
Inés	Legarda Ramírez	Hospital Universitario Son Espases
Bàrbara	Vives Pastor	Hospital Universitario Son Espases
María Fuensanta	Valero García	Hospital Universitario Son Espases
Esther	Cubo Delgado	Hospital Universitario de Burgos
Nuria	López Ariztegui	Hospital Universitario de Toledo
Maria Isabel	Morales Casado	Hospital Universitario de Toledo
Guillermo	Tabar Comellas	Hospital Universitario de Toledo
Déborah	Alonso Modino	Hospital Universitario de la Candelaria
Jesús Norelis	Lorenzo Brito	Hospital Universitario de la Candelaria
María Esther	Rojas Pérez	Hospital Universitario de la Candelaria
Carolina	Hernández Javier	Hospital Universitario de la Candelaria
Iria	Cabo López	Complejo Hospitalario Universitario de Pontevedra
Alejandro	Rivero de Aguilar Pensado	Complejo Hospitalario Universitario de Pontevedra
Jorge	Hernández Vara	Hospital Universitario Vall d’Hebron
Maria Victoria	González	Hospital Universitario Vall d’Hebron
Sara	Belmonte ^1^	Hospital Universitario Vall d’Hebron
Juan Carlos	Romero Fábrega	Hospital Universitario Virgen de las Nieves
Francisco	Escamilla Sevilla	Hospital Universitario Virgen de las Nieves
Lucía	Triguero Cueva	Hospital Universitario Virgen de las Nieves
Carlos Javier	Madrid Navarro	Hospital Universitario Virgen de las Nieves
Rosa	Yáñez Baña	Complejo Hospitalario Universitario de Ourense
Asunción	Ávila Rivera	Complex Hospitalari Moisès Broggi
Núria	Caballol Pons	Complex Hospitalari Moisès Broggi
Anna	Planas-Ballvé	Complex Hospitalari Moisès Broggi
Alejandro	Peral Quirós	Complex Hospitalari Moisès Broggi
Dolors	Lozano	Complex Hospitalari Moisès Broggi
Álvaro	Sánchez Ferro	Hospital 12 de Octubre
Pablo	Rábano Suárez	Hospital 12 de Octubre
Antonio	Méndez Guerrero	Hospital 12 de Octubre
Daniel	Pérez Rangel	Hospital 12 de Octubre
Jesús	Ramírez	Hospital 12 de Octubre
Cristina	del Álamo Criado	Hospital 12 de Octubre
Rocío	García Ramos	Hospital Clínico Universitario San Carlos
Ana	Fernández Revuelta	Hospital Clínico Universitario San Carlos
Eva	López Valdés	Hospital Clínico Universitario San Carlos
Carmen	Ribacoba Díaz	Hospital Clínico Universitario San Carlos
Ana	Aldaz	Hospital Clínico Universitario San Carlos
Irene	Martínez-Torres	Hospital Universitario la Fe
Carlos	Morata-Martínez	Hospital Universitario la Fe
Raquel	Baviera-Muñoz	Hospital Universitario la Fe
Marina	Campins Romeu	Hospital Universitario la Fe
Isabel	Sastre Bataller	Hospital Universitario la Fe
Elena	Navarro Mocholi	Hospital Universitario la Fe
Pilar	Sánchez Alonso	Hospital Puerta de Hierro
Sabela	Novo Ponte	Hospital Puerta de Hierro
Elisa	Gamo González	Hospital Puerta de Hierro
Raquel	Martín García	Hospital Puerta de Hierro
Pablo	Mir Rivera	Hospital Universitario Virgen del Rocío
Laura	Muñoz Delgado	Hospital Universitario Virgen del Rocío
Astrid Daniela	Adarmes Gómez	Hospital Universitario Virgen del Rocío
Elena	Ojeda Lepe	Hospital Universitario Virgen del Rocío
Silvia	Jesús Maestre	Hospital Universitario Virgen del Rocío
Daniel	Macías García	Hospital Universitario Virgen del Rocío
Fátima	Carrillo García	Hospital Universitario Virgen del Rocío
Ana María	Castellano Guerrero ^2^	Hospital Universitario Virgen del Rocío
Manuela	San Eufrasio Martínez ^2^	Hospital Universitario Virgen del Rocío
Cristina	Pérez Calvo ^2^	Hospital Universitario Virgen del Rocío
Andrés	García Herruzo	Hospital Universitario Virgen del Rocío
Cristina	Gómez Rapela ^3^	Hospital Universitario Virgen del Rocío
Lorena	Garrote Espina ^3^	Hospital Universitario Virgen del Rocío
Natalia	González Rojas ^3^	Hospital Universitario Virgen del Rocío
Marta	Blázquez Estrada	Hospital Universitario Central de Asturias
Esther	Suárez San Martín	Hospital Universitario Central de Asturias
Ciara	García Fernández	Hospital Universitario Central de Asturias
Patricia	Prendes Fernández ^4^	Hospital Universitario Central de Asturias
Pilar	Tartiere ^4^	Hospital Universitario Central de Asturias
Pedro	García Ruíz Espiga	Hospital Fundación Jiménez Díaz
Cici	Feliz Feliz	Hospital Fundación Jiménez Díaz
Raúl	Espinosa Rosso	Hospital Universitario de Jerez
Lara	Mauri Fabrega	Hospital Universitario de Jerez
Marina	Mata Álvarez-Santullano	Hospital Universitario Infanta Sofía
Juan Carlos	Gómez Esteban	Hospital de Cruces
Tamara	Fernández Valle	Hospital de Cruces
Marta	Ruiz López	Hospital de Cruces
Beatriz	Tijero Merino	Hospital de Cruces
Lydia	López Manzanares	Hospital Universitario La Princesa
Inés	Muro	Hospital Universitario La Princesa
Elena	Casas Peña	Hospital Universitario La Princesa
Pablo	Lorenzo Barreto	Hospital Universitario La Princesa
Emma	Cañada Lahoz	Hospital Universitario La Princesa
Sara	Lozano Veiga	Hospital Universitario La Princesa
Sara	García Cimadevilla	Hospital Universitario La Princesa
Carmen	Martínez Miguel	Hospital Universitario La Princesa
Raquel	Marcos Conca	Hospital Universitario La Princesa
Maria Pilar	Gil Villar	Hospital Universitari Arnau de Vilanova
Agustín	Sancho Saldana	Hospital Universitari Arnau de Vilanova
Laura	Quibus Requena	Hospital Universitari Arnau de Vilanova
Víctor	Gómez Mayordomo	Hospitales Vithas
Berta	Solano Vila	Hospital Josep Trueta de Girona y Hospital Santa Caterina de Salt
Anna	Cots Foraster	Hospital Josep Trueta de Girona y Hospital Santa Caterina de Salt
Daniel	López Dominguez	Hospital Josep Trueta de Girona y Hospital Santa Caterina de Salt
Lilian	Vivas Villacampa	Hospital Josep Trueta de Girona y Hospital Santa Caterina de Salt
Jessica	González Ardura	Hospital de Cabueñes
Belén	Castaño García	Hospital de Cabueñes
Antonio	Sánchez Rodríguez	Hospital de Cabueñes
Sonia	Escalante Arroyo	Hospital Virgen de la Cinta
Marcos Alfredo	Mandrá	Hospital Virgen de la Cinta
Tania	Delgado Ballestero	Parc Taulí
Débora María	Cerdán Santacruz	Hospital General de Segovia
Amelia	Mendoza Rodríguez	Hospital General de Segovia
Ana	Castrillo Sanz	Hospital General de Segovia
Lorena	Caballero Sánchez	Hospital General de Segovia
Claudia	Gómez López de San Román	Hospital General de Segovia
Gustavo	Fernández-Pajarín	Complejo Hospitalario Universitario de Santiago de Compostela
Susana	Cantarero Duque	Hospital Universitario de Móstoles
Guillermo	González-Ortega	Hospital Universitario de Móstoles
Silvia	Martí Martínez	Hospital General Universitario Alicante
Lydia	Vela	Hospital Fundación de Alcorcón
Javier	Ruíz Martínez	Hospital Universitario Donostia
Elisabet	Mondragón Rezola	Hospital Universitario Donostia
Ana	Vinagre Aragón	Hospital Universitario Donostia
Lara	Pardina Vilella	Hospital Universitario Donostia
David	Campo Caballero	Hospital Universitario Donostia
Roberto	López Blanco	Hospital Universitario Severo Ochoa, Leganés, Madrid
Carolina	Hernández Javier	Hospital Universitario de Canarias (HUC), Santa Cruz de Tenerife.
	The researchers are listed by center in chronological order according to the date they confirmed their participation in the project. All investigators are neurologists except those ones with the symbol: ^1^ SN (specialized nurse); ^2^ graduate in Biology (she will be responsible for remote data monitoring); ^3^ neuropsychologist; ^4^ study coordinator.	
